# Evaluation and assessment of the wettabilty and water contact angle of modified poly methyl methacrylate denture base materials against PEEK in cast partial denture framework: an in vitro study

**DOI:** 10.1186/s12903-023-03716-2

**Published:** 2024-02-17

**Authors:** Joshua Narde, Nabeel Ahmed, Yuliia Siurkel, Maria Maddalena Marrapodi, Vincenzo Ronsivalle, Marco Cicciù, Giuseppe Minervini

**Affiliations:** 1 Saveetha Dental College and Hospitals, Saveetha Institute of Medical and Technical Sciences (SIMATS), Saveetha University, Chennai, Tamil Nadu India; 2 Multidisciplinary Department of Medical-Surgical and Dental Specialties, University of Campania “Luigi Vanvitelli”, Naples, 80121 Italy; 3 Department of Prosthodontics, Saveetha Institute of Medical and Technical Sciences, Saveetha Dental College and Hospital, Saveetha University, Chennai, India; 4 International European University School of Medicine, Akademika Hlushkova Ave, 42B, Kyiv, 03187 Ukraine; 5 Department of Woman, Child and General and Specialist Surgery, University of Campania “Luigi Vanvitelli”, Naples, 80121 Italy; 6 Department of Biomedical and Surgical and Biomedical Sciences, Catania University, Catania, 95123 Italy

**Keywords:** Partial denture [MeSH terms], PEEK, Biodentaplast, Polyan, Contact angle, Wettability [MeSH terms]

## Abstract

**Introduction:**

The prevalence of adults with partially dental arches is expected to be more than imagined and patients requiring replacement of missing teeth are slowly increasing in number too. Removable partial dentures are known to provide for substantial replacement for the missing teeth with also added advantages when compared to fixed or implant prosthesis, mainly in elderly patients. Denture base material performance and durability are greatly influenced by wettability and water contact angle. In the case of dentures; adequate moisture distribution is necessary to ensure excellent wettability which has an influence on comfort and oral health. The purpose of conducting this study was to find out whether the advancements made using PEEK (Polyether ether ketone) would prove to be more beneficial than the current upgrades in the current material spectrum.

**Materials and methods:**

This study was performed under in vitro conditions. All the fabrication and processing was done only by one operator. The materials used were divided into three groups each comprising 20 samples. Group A was modified polymethylmethacrylate (Bredent Polyan), Group B was polyoxymethylene acetal resin (Biodentaplast) and Group C was PEEK. An Ossila Goniometer was used to measure the contact angle. The three types of liquids used for the testing included distilled water, natural saliva and mouth wetting solution (Wet Mouth Liquid, ICPA India). Human saliva was collected from an individual with no medical conditions and normal salivary secretion.

**Results:**

The data was analyzed using One-way ANOVA test and a pairwise comparison using the Post Hoc Tukey’s Honest Significant Difference. Table 1 consists of the mean water contact angles of the denture base materials and mean contact angles of various denture base materials. In saliva, mouth wetting solution and distilled water, the highest mean and least mean contact angle was seen in Polyan and Biodentaplast respectively. A signicant difference was seen between PEEK and Polyan and Biodentaplast and Polyan on further comparison.

**Conclusion:**

From the resources and the materials at our disposal, it could be concluded that Polyan, Biodentaplast and PEEK and could be used as viable options in cast partial denture framework.

## Introduction

The prevalence of adults with partially dental arches is expected to be more than imagined and patients requiring replacement of missing teeth are slowly increasing in number too [1, 2]. Among the options available, removable partial dentures are known to provide for substantial replacement for the missing teeth with also added advantages when compared to fixed or implant prosthesis, mainly in elderly patients [3]. A variety of materials are available for the manufacturing of a cast partial denture. Traditionally these dentures are made from casting of a wax framework [4] but with the advent of computer aided design and computer aided manufacturing(CADCAM), these prosthesis can now be designed and delivered using digital techniques too [5]. The metal framework could be produced using either cobalt chromium alloys or even titanium on which conventional acrylic material could be used. Acrylic could also be replaced by newer materials as thermoplastic acrylic, polyoxymethylene, PEEK and even flexible resins. Denture base material performance and durability are greatly influenced by wettability and water contact angle. The ability of a material to spread and cling to saliva or water is referred to as wettability [6, 7]. In the case of dentures, adequate moisture distribution is necessary to ensure excellent wettability, which in turn influences comfort and oral health. Better wettability, or the ability of a material to absorb moisture more easily, is indicated by a lower water contact angle. This is crucial since tissue irritation and oral health problems can be brought on by dryness.

With the increase in allergies seen in patients to the classic polymethylmethacrylate materials [8, 9] many dentists now opt for presumably hypoallergenic denture base resins that include polyurethane, polyethylenterephthalate, polybutylenterephthalate and modified methacrylate based denture bases [10]. Polyan is considered to be a thermoplastic modified methacrylate product which displayed less residual monomer content [11–23].

Since 1986, polyoxymethylene (POM), commonly known as acetal resin, has been used as an alternate denture foundation and denture clasp material, largely to improve aesthetics [24]. An example of this material would be Biodentaplast. Acetal resins have been employed as a denture base and clasp material as an alternative [25]. They are made from formaldehyde polymerization and have been proposed as an alternate material for removable denture framework production in individuals who have allergies to cobalt–chromium alloys [24]. It has enough resilience and modulus of elasticity, as well as high impact strength and resistance to organic solvents, oils, and hot and cold water [26], to be used in the fabrication of frameworks, retentive clasps, connectors, and support elements for removable partial dentures [26]. Another well-known high-performance thermoplastic aromatic polymer is poly ether-ether-ketone (PEEK). It’s a two-phase, semicrystalline polymer with a crystallinity of 30 to 35%, depending on the manufacturing method [27]. It has good mechanical qualities as well as excellent biocompatibility. It is thought to be a good material for removable denture frameworks and their components, including esthetic clasps [28–30].

The purpose of conducting this study was to find out whether the advancements made using PEEK would prove to be more beneficial than the current upgrades in the current material spectrum. According to the authors knowledge no such study or research has been conducted before comparing the efficacy of these three materials specifically.

The null hypothesis is that modified methacrylate, polyoxymethylene and polyetheretherketone(PEEK) all have the same physical properties when used in the fabrication of removable partial dentures.

## Materials and methods

This study was performed under in vitro conditions. All the fabrication and processing was done only by one operator. The materials used were divided into three groups. Group A was modified polymethylmethacrylate (Bredent Polyan), Group B was polyoxymethylene acetal resin (Biodentaplast) and Group C was Polyethertetherketone (PEEK).

The three types of liquids used for the testing included distilled water, natural saliva and mouthwetting solution(Wet Mouth Liquid, ICPA India). The study was conducted in accordance with the Declaration of Helsinki, and the protocol was approved by the Ethics Committee of the Institute, Saveetha Dental College And Hospitals [Protocol number: SRB/SDC/PROSTHO-2102/22/105; Date: 09/08/2022]. The study protocol was developed, and all subjects gave their written informed consent for inclusion before they participated in the study.

### Sample fabrication

#### Polyan

**Fig. 1:**
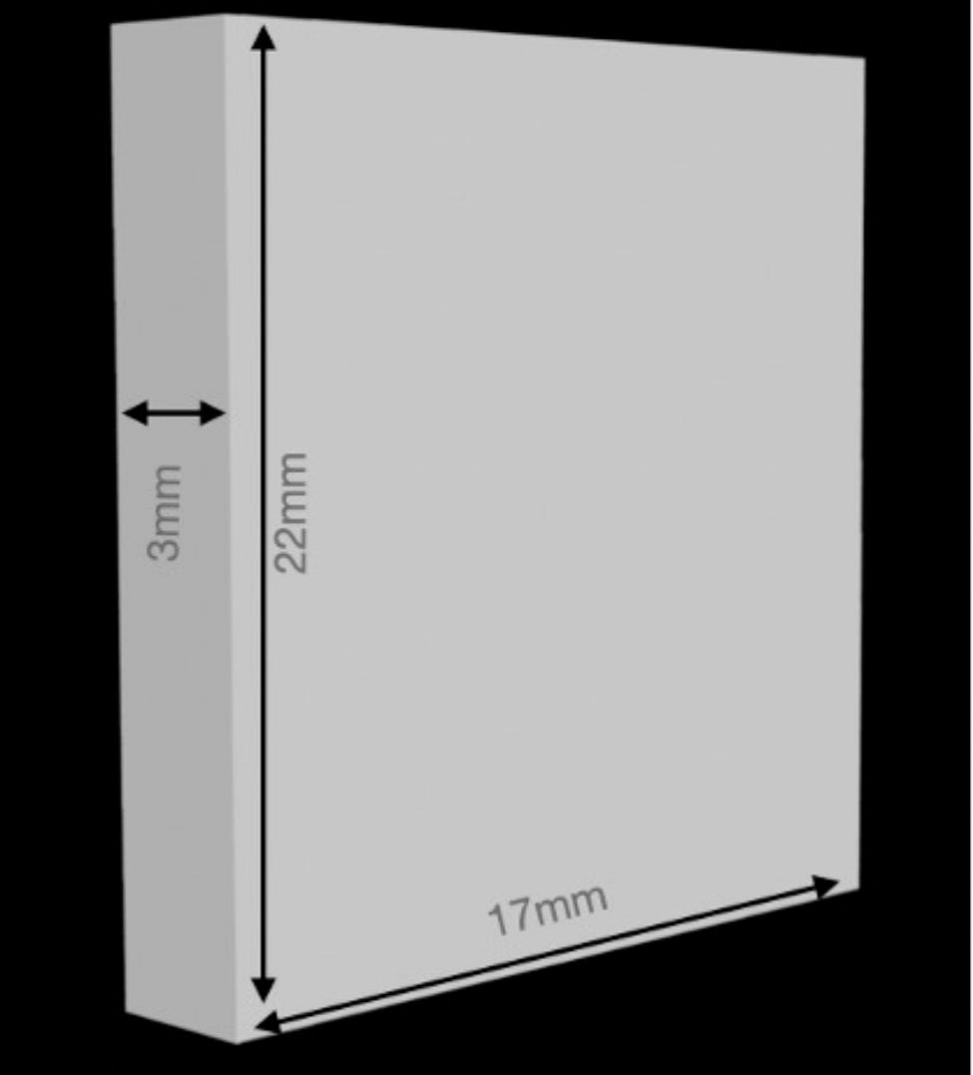
The dimensions of the designed STL file to be milled

**Fig. 2:**
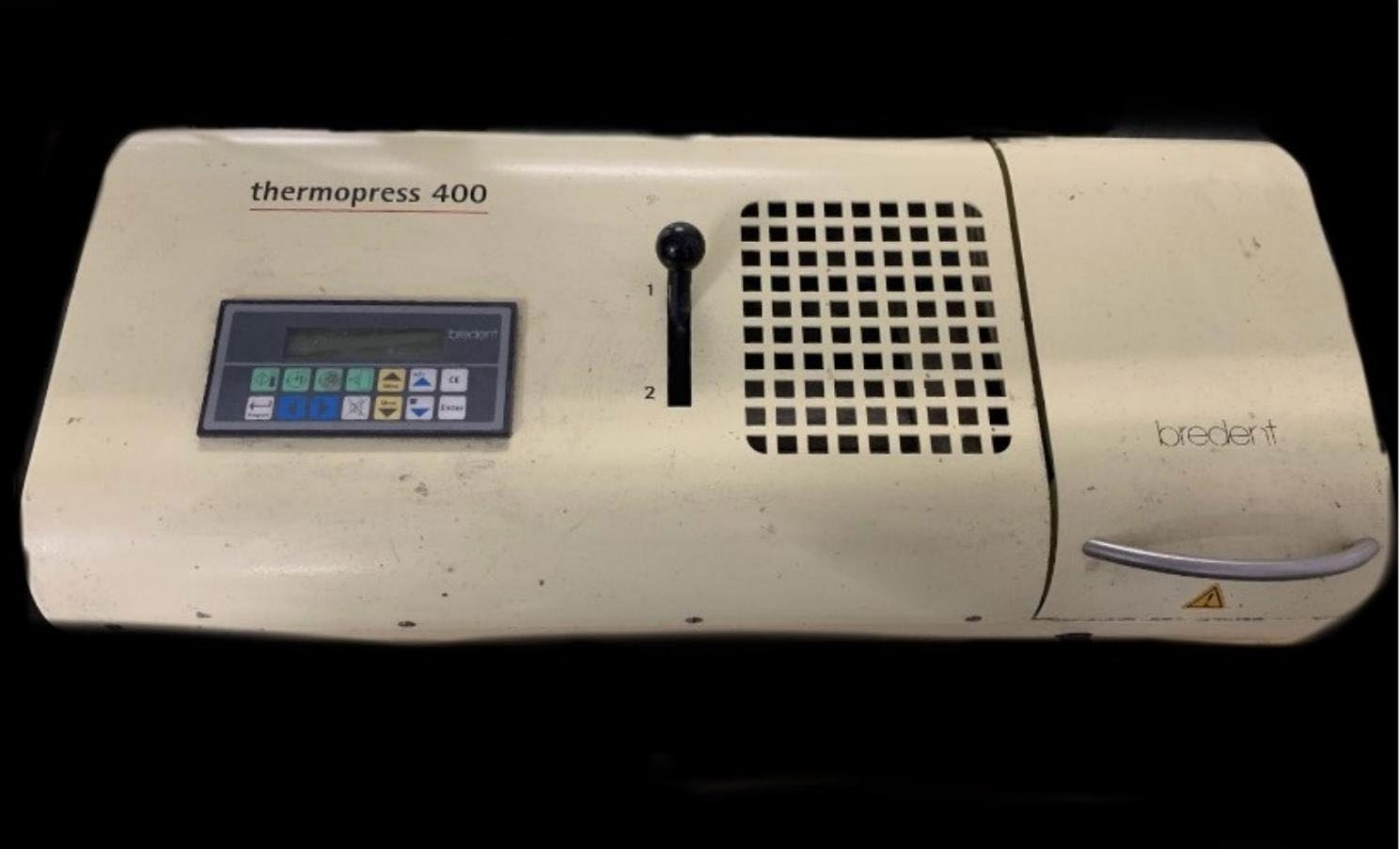
Thermopress 400

20 wax patterns of the dimensions 22 mm x 17 mm x 3 mm were fabricated. An STL file (Fig. 1) was created of the correct dimensions and the wax was milled into 3 specimens (Roland DGShape, DWX 52D). This was done to avoid any error in the thickness of the slice obtained. Once fabricated, they were then invested in a flask specifically for this material after which dewaxing was carried out. A film sprue is attached to the wax model to benefit from the mechanical properties of the thermoplastics. Sprue application is done using a 1.5 mm thick wax plate. A 10 mm thick injection moulding sprue directly to the model with the help of a 1.5 mm wax plate. Polyan material was then loaded into an aluminum cartridge. The processing is carried out in Thermopress 400 (Fig. 2) injection moulding device with the processing parameters already present in the device as per the manufacturers recommendations. The surface which acts as the tissue surface is not polished. A tungsten carbide bur was used to remove extra resin from the specimens before finishing them with wet silicon carbide sheets (600-grit, 800-grit, 1000-grit, and 1200-grit). Only one surface was wet in order to simulate laboratory techniques and it was polished with a cloth wheel and pumice. A final size of 20 mm x 15 mm x 2 mm was obtained. Each Polyan slice was then used for one media only.

#### Biodentaplast

20 wax patterns of the dimensions 22 mm x 17 mm x 3 mm were fabricated. The same design was used for the creation of the wax pattern. Once obtained, special flasks were used to carry out the investing followed by the dewaxing. A sprue was attached to achieve the benefits of the thermoplastic materials. The ‘Thermopress 400’ machine was used for the processing as per the manufacturers recommendations. Polishing was done on only one surface that would act as the denture base area. Each Biodentaplast slice was then used for one media only.

#### Peek

20 rectangular slices of dimensions 22 mm x 17 mm x 3 mm were first milled in wax. The slices were then placed in a flask and dewaxing was carried out after which they were placed in the Thermopress 400 and processed based on the preset settings. After processing and polishing, each obtained PEEK slice was used for one media.

### Wettability and contact angle

**Fig. 3:**
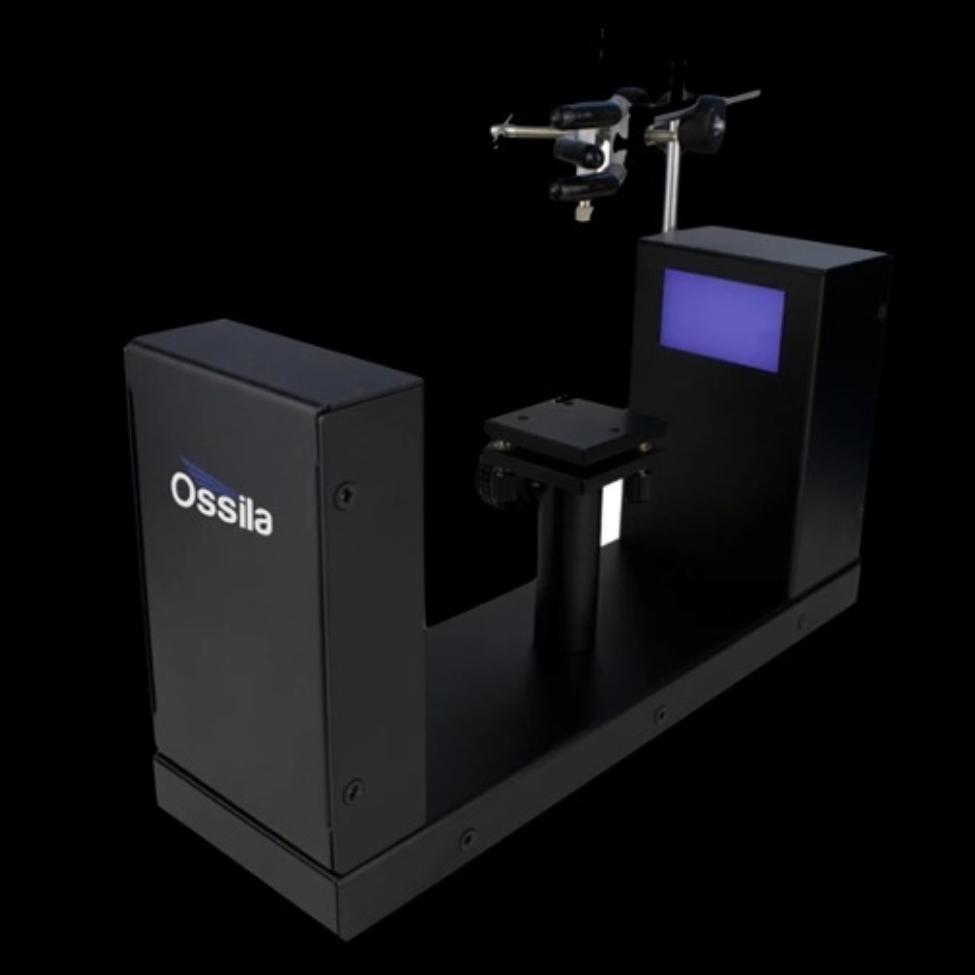
Ossila Goniometer

A micropipette was used and a drop of media in a volume of 1.0 L, the sessile drop method was used to calculate the static contact angle. Within 2 s of placing the media on the surface of the specimen, a set of 3 photos was taken, and the contact angle was then determined using the Ossila contact angle goniometer (Fig. 3) and axisymmetric drop shape analysis. Getting a picture of a droplet on a flat surface is the first stage in the measurement. The baseline of the droplet is manually designated at the intersection of the real image and its reflection after the droplet on the flattened specimen has been captured in an image.

The contact angle goniometer software programmatically marks the tracing of the droplet edge and the gradient of the tangent of the droplet edge to the point where it meets the baseline, and then calculates the contact angle between them on the left and right sides of the sample. All of the samples’ contact angles were measured and the baseline of the droplet inside the field of interest was marked by a single operator. The same observer again measured the sessile drop contact angle for all the remaining samples. Care was taken to completely rinse the dispensing syringe with water before adding the fluid that would be analyzed to the specimen.

The groups were divided in the following manner:

#### Group A

1. Saliva and Polyan.
2. Mouth wetting solution and Polyan.
3. Distilled water and Polyan.

#### Group B

1. Saliva and Biodentaplast.
2. Mouth wetting solution and Biodentaplast.
3. Distilled water and Biodentaplast.

#### Group C

1. Saliva and PEEK.
2. Mouth wetting solution and PEEK.
3. Distilled water and PEEK.

To avoid any error, each media was applied thrice on the specimen. In total 9 groups were formed.

Statistical analysis was done using IBM SPSS Software (version 23).

## Results

The data was analyzed using One-way ANOVA test and a pairwise comparison using the Post Hoc Tukey’s Honest Significant Difference.

Table 1 depicts the mean contact angles of various denture base materials. In saliva, mouth wetting solution and distilled water, the highest mean and least mean contact angle was seen in Polyan and Biodentaplast respectively.

**Table 1:** Mean contact angle of the DBMs in different solutions

SOLUTION	DBM	Mean	Std. Deviation	Minimum	Maximum
SALIVA	PEEK	71.4667	6.73914	63.83	76.58
	POLYAN	92.2133	6.61158	87.00	99.65
	BIODENTAPLAST	59.8767	3.58984	56.05	63.17
	Total	74.5189	15.06006	56.05	99.65
MW	PEEK	72.1333	14.34481	55.76	82.49
	POLYAN	83.0300	2.31715	71.25	75.65
	BIODENTAPLAST	59.4967	20.74487	44.82	83.23
	Total	68.2200	14.25932	44.82	83.23
DW	PEEK	59.7433	5.87746	53.45	65.09
	POLYAN	78.510	7.47115	47.74	60.74
	BIODENTAPLAST	52.113	19.41509	57.91	96.47
	Total	63.4556	15.97578	47.74	96.47

In saliva, a significant difference in the contact angle was seen between the three different materials (*p* = 0.001). However with respect to distilled water and mouth wetting solution, no significant difference was observed between the three materials (*p* > 0.05). [Table 2]

**Table 2:** Comparison of mean contact angle between the three different DBMs using One way ANOVA test

SOLUTION	DBM	F	Sig
Saliva	PEEK POLYAN BIODENTAPLAST	23.679	0.001
MW	PEEK POLYAN BIODENTAPLAST	0.804	0.491
DW	PEEK POLYAN BIODENTAPLAST	3.554	0.096

**Fig. 4:**
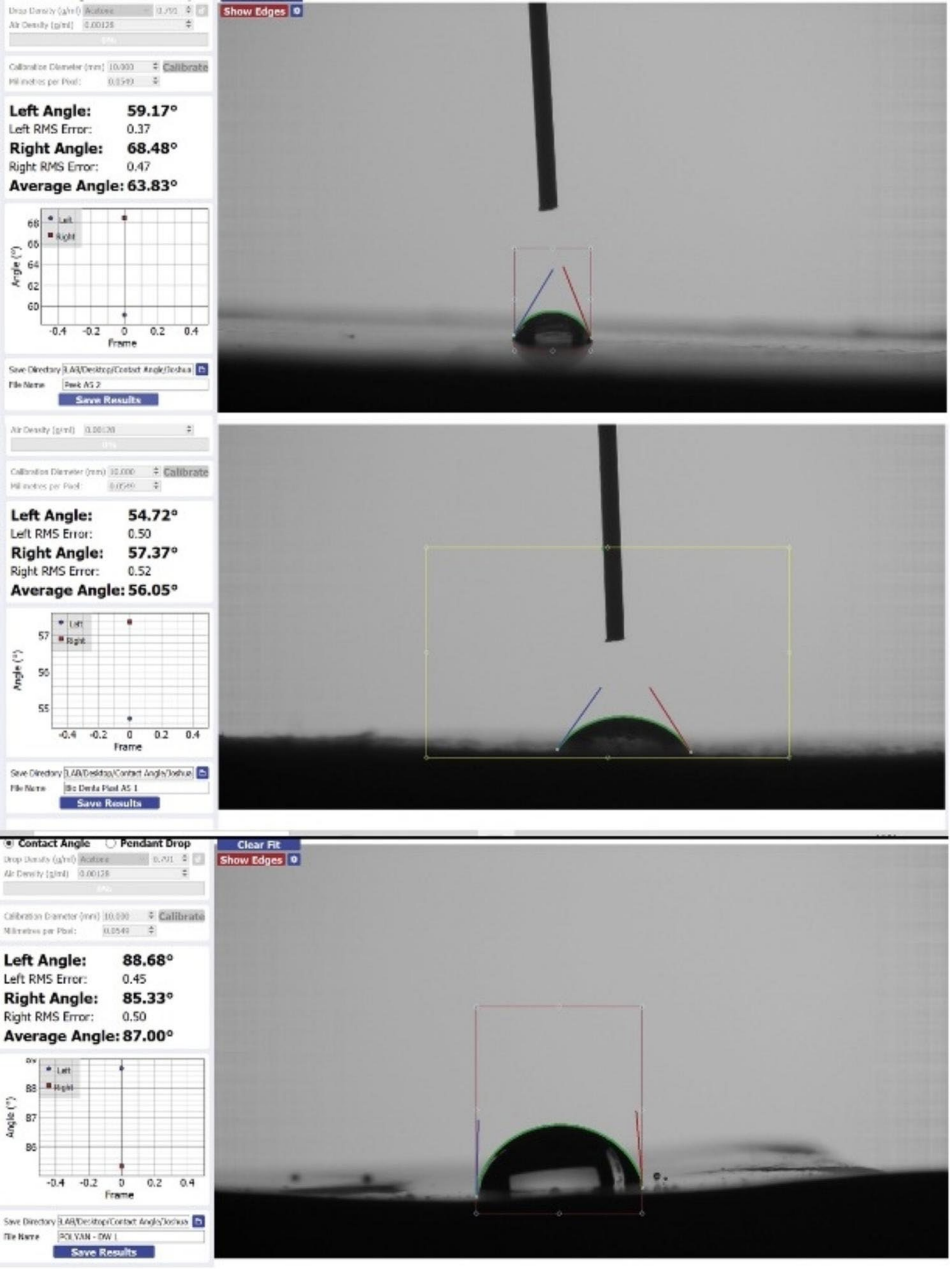
Contact angle measured for Polyan, Biodentapalst and PEEK

**Fig. 5:**
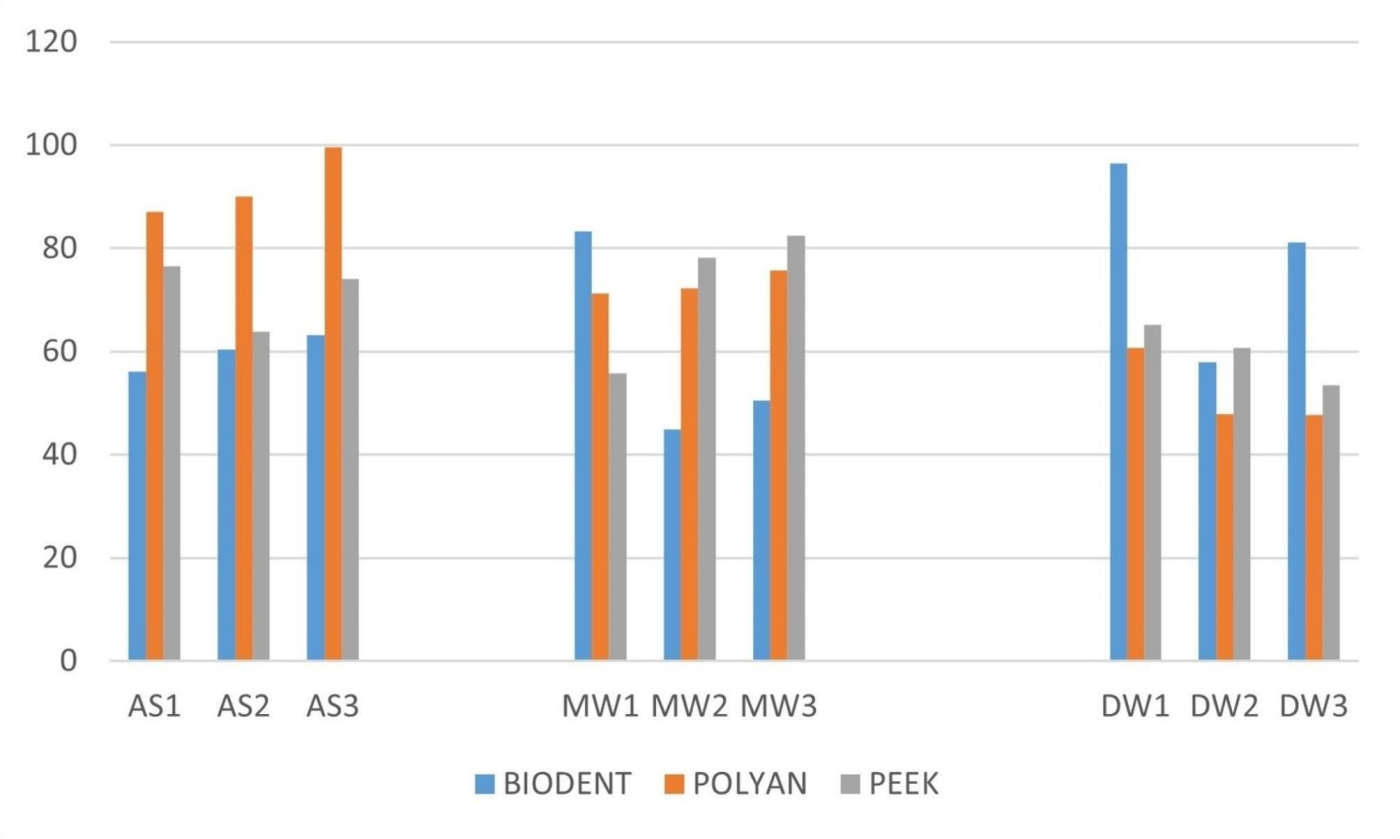
The contact angles for the three materials when tested with Saliva, Mouth wetting solution and distilled water

Table 3 depicts the pairwise comparisons between the DBMs in saliva. A significant difference was observed between the mean contact angle of PEEK and Polyan, as well as between Polyan and Biodentaplast. However, no significant difference was seen between PEEK and Biodentaplast. Polyan exhibited the highest contact angle followed by PEEK and Biodentaplast (Figs. 4 and 5).

**Table 3:** Pairwise comparison of mean contact angle between the DBMs in saliva using Post hoc Tukey’s HSD test

Medium	(I) DBM	(J) DBM	Mean Difference (I-J)	Std. Error	Sig.
ARTIFICIAL SALIVA	PEEK	POLYAN	-20.74667^*^	4.76133	0.011
		BIODENTAPLAST	11.59000	4.76133	0.111
	POLYAN	PEEK	20.74667^*^	4.76133	0.011
		BIODENTAPLAST	32.33667^*^	4.76133	0.001
	BIODENTAPLAST	PEEK	-11.59000	4.76133	0.111
		POLYAN	-32.33667^*^	4.76133	0.001

## Discussion

Denture base materials with superior wettability help maintain a moist oral environment, preventing dryness, discomfort, and irritation for the wearer. This is particularly important as dryness can lead to tissue inflammation and oral health issues. On the other hand, poor wettability can result in water droplets forming on the surface, making dentures uncomfortable and potentially affecting speech and mastication [31]. Denture base materials’ performance and patient satisfaction are significantly influenced by the contact angle. The capacity of a material to moisten or spread across a surface is determined by the contact angle. It can also contribute to bacterial colonization, leading to hygiene concerns. Additionally, it may restrict normal speech and mastication, making it difficult for the wearer to properly talk and eat. Materials with favorable wettability characteristics contribute to better patient satisfaction and oral well-being.

The forces required to completely remove the denture from its basal seat are related to denture retention [32]. Wetting of the denture and palate via the respective adhesive forces at the two interfaces is required prior to retention [33]. The retention force, according to Stanitz, is a function of saliva surface tension, liquid film thickness, surface of contact, and liquid-denture contact angle [34].

With the exception of some specific cases, such as perfectly wettable solids, theoretical considerations and experimental results have demonstrated that the contact angle of the advancing liquid front on a dry solid surface (advancing contact angle A) differs from the receding contact angle (R) formed when the liquid recedes on a previously wet surface [35, 36]. In this study, the technique of the goniometer involves the use of the sessile drop. The measurement of the right and left contact angle which then gives an average contact angle measurement [37]. The contact angle which is > 90° leads to the formation of droplets of the liquid over the surface of the denture base material hence rendering it to be hydrophobic. The contact angle when measured to be < 90° causes uniform distribution of the liquid over the surface of the denture base material and that is what leads to increased wettability. This angle is formed is formed at an intersection interface of solid, liquid and gas. With the increase in the wettability; there is an increase in the retention of the denture base to the oral tissues. High contact angles can make the denture base materials reject saliva while allowing water droplets to condense on the surface [37]. This could lead to hygienic problems and intraoral infections by the formation of retention sites for bacteria and debris. Low contact angle materials, on the other hand, encourage uniform moisture dispersion, which can support improved oral hygiene. According to Monsenego et al., in his in vitro study, the most convenient denture base material would be one with the highest contact angle hysteresis, such as a high advancing contact angle and a low receding contact angle [32, 38]. Waters et al. came to the conclusion that higher contact angle hysteresis values of soft-lining denture materials compared to polymethylmethacrylate denture base material indicated that all soft lining materials would improve denture stability under dislodgement forces [39].

In the study conducted, significant difference was only noted in the among the groups when saliva was the liquid in contact. PEEK and Biodentplast showed no significant difference between them whereas there was a difference that was noted among the PEEK and Polyan group and the Biodentaplast and Polyan group. Polyan displayed the highest mean contact angle (92.2133) which signifies an increased contact angle and thus reduced wettability. The specimens of Biodentaplast and PEEK on the other hand displayed a lower mean contact angle when tested with the saliva sample. Biodentaplast was developed as a material to overcome the challenges that were noted by the conventional acrylic resins. A low modulus of elasticity, lack of reactivity to different solutions, good polishability, a proven tensile and mechanical strength, good colour properties and non allergic makes Biodentaplast a suitable option for the cst partial denture framework [40]. Due to its lack of free energy on the surface, it also attracts less bacteria in the oral cavity. It is retentive when used as a framework material and harbours less microorganisms when used as a clasp material. PEEK is a high performance polymer which has been developed to be used as a multipurpose material in dentistry. Its use in esthetic clasp partial dentures has now become popular due to good surface properties, strength and the sufficient esthetic advantage [41–43].

Various surface properties also play a key role in the wettability and contact angle of denture base materials. Factors such as the heterogeneity, polishing properties, roughness, deformation and adherence to other substances are noted to alter the wettability(Bin et al., 2017). The presence of an increased surface energy can result in the quicker formation of a biofilm and a lack of retention. The materials that were selected had similar properties when they were placed in both the solutions. This observation can be correlated from a study conducted by Ramanna [44]. The mouth wetting solutions that are commercially available are of two types: mucin based and carboxymethylcellulose(CMC) based. Studies have gone to show that both these substitutes are effective in cases of dry mouth in denture wearers and reduce discomfort that could be associated(Bin et al., 2017). The contact angle of that of saliva and these substitutes are nearly similar with most materials.

Choosing a denture base material with good wettability can lead to an increase in the comfort to the tissues and oral health, good aesthetics, better masticatory performance, and longevity. The increased wettability makes it less susceptible to contamination and degradation over the long run.

The limitations of the study include the use of only a certain materials and not all the materials that could be incorporated as cast partial denture framework materials. The use of only a CMC solution and not a mucin based one could also lead to certain changes. More clinical trials are required for the same.

## Conclusion

From the resources and the materials at our disposal, it could be concluded that Polyan, Biodentaplast and PEEK and could be used as viable options in cast partial denture framework. PEEK and biodnetpalast showed almost similar readings compared to Polyan which showed lesser properties compared to the other two materials and a significant difference was noted in saliva and mouth wetting solutions. Hence the null hypothesis was rejected. Although more research and clinical trials are needed to prove the same.

## Data Availability

The data will be available on reasonable request from the corresponding author.

## List of abbreviations

DW: Distilled water
MW: Mouth wetting solution
PEEK: Polyether ether ketone
